# An autism-causing calcium channel variant functions with selective autophagy to alter axon targeting and behavior

**DOI:** 10.1371/journal.pgen.1008488

**Published:** 2019-12-05

**Authors:** Tyler Buddell, Vladislav Friedman, Cody J. Drozd, Christopher C. Quinn

**Affiliations:** Department of Biological Sciences, University of Wisconsin-Milwaukee; Milwaukee, Wisconsin, United States of America; Columbia University Medical Center, UNITED STATES

## Abstract

Common and rare variants of the *CACNA1C voltage-gated calcium channel* gene have been associated with autism and other neurodevelopmental disorders including schizophrenia, bipolar disorder and ADHD. However, little is known about how *CACNA1C* variants affect cellular processes to alter neurodevelopment. The Timothy syndrome mutation is a rare *de novo* gain-of-function variant in *CACNA1C* that causes autism with high penetrance, providing a powerful avenue into investigating the role of *CACNA1C* variants in neurodevelopmental disorders. Here, we use *egl-19*, the *C*. *elegans* homolog of *CACNA1C*, to investigate the role of voltage-gated calcium channels in autism. We show that an *egl-19(gof)* mutation that is equivalent to the Timothy syndrome mutation can alter axon targeting and affect behavior in *C*. *elegans*. We find that wildtype *egl-19* negatively regulates axon termination. The *egl-19(gof)* mutation represses axon termination to cause axon targeting defects that lead to the misplacement of electrical synapses and alterations in habituation to light touch. Moreover, genetic interactions indicate that the *egl-19(gof)* mutation functions with genes that promote selective autophagy to cause defects in axon termination and behavior. These results reveal a novel genetic mechanism whereby a *de novo* mutation in *CACNA1C* can drive alterations in circuit formation and behavior.

## Author summary

Autism is a disorder that affects neuronal development, leading to alterations in cognition and behavior. Imaging studies have revealed alterations in axonal connectivity as a key feature of autism. However, the underlying perturbations in cell biology that drive these alterations remain largely unknown. To address this issue, we have taken advantage of the Timothy syndrome mutation, a variant in a voltage-gated calcium channel that has the unusual property of causing autism with high penetrance. We identify a role for wild-type voltage-gated calcium channels in regulating axon targeting in *C*. *elegans*. Moreover, we find that two different versions of the Timothy syndrome mutation disrupt axon targeting. Our results suggest that the Timothy syndrome mutations disrupt axon targeting and behavior by interacting with genes that promote selective autophagy, the process through which cellular components are selected for degradation. These results reveal a mechanism through which variants in voltage-gated calcium channels can cause the disruptions in axonal connectivity that underlie autism.

## Introduction

Variants in the *CACNA1C* voltage-gated calcium channel (VGCC) gene are common risk factors for autism and other neurodevelopmental disorders including schizophrenia, bipolar disorder and attention deficit hyperactivity disorder (ADHD). For example, genome wide association studies (GWAS) have associated common variants in *CACNA1C* to autism [1, 2]. Moreover, statistical analysis of whole genome sequencing data indicates that rare variants in *CACNA1C* are also associated with autism [3–10]. Whereas the evidence is strongest for *CACNA1C*, variants in other VGCC subunit genes are also associated with autism [2, 8, 11, 12]. Despite these insights from statistical analysis, little is currently known about how variants in VGCC genes affect cellular processes to disrupt neurodevelopment.

A major impediment to understanding how autism-associated variants affect cellular processes is that most variants have a small effect size. Because each variant only has a small effect, it is thought that multiple variants engage in genetic interactions that give rise to the neurodevelopmental defects underlying autism [13, 14]. Therefore, a key goal in understanding the biological basis for autism is to understand genetic interactions between autism-associated variants. However, currently little is known about how autism-associated variants interact with each other. Moreover, in most cases the cellular mechanisms perturbed by each variant are also unknown.

Morphological abnormalities in axon development are associated with autism and other neurodevelopmental disorders [15–19]. Most mechanistic studies of autism-associated genes have focused on dendrite and synapse structure. However, imaging studies have suggested that alterations in axon targeting are a key feature of autism. For example, diffusion tensor imaging has revealed alterations in the Inferior Longitudinal Fasciculus in autistic individuals relative to healthy controls [15–19]. Moreover, functional MRI has revealed alterations in long range connectivity that can predict autism in individuals before the onset of symptoms [20–22]. These observations suggest that alterations in axon targeting are likely to underlie autism. However, little is currently known about how autism-associated variants can alter axon targeting.

In this work, we use the Timothy syndrome mutation as a platform to discover how autism-associated variants interact with each other to alter cellular processes and disrupt axon development. The Timothy syndrome mutation is actually a class of three rare *de novo* mutations in *CACNA1C* that encode either a G402R, G402S or G406R mutation in the CACNA1C protein. [10, 23]. Although the Timothy syndrome mutation is rare with large effect, common variants with small effect in *CACNA1C* are also associated with autism [1, 2]. As the mechanisms by which CACNA1C affects axon development are unknown, studies of the Timothy Syndrome variant may uncover genetic mechanisms that also apply to the more common *CACNA1C* risk variants.

Here, we investigate the role of *egl-19*, the *C*. *elegans* homolog of *CACNA1C*, in axon targeting. Like CACNA1C, EGL-19 is the pore-forming subunit for L-type voltage-gated calcium channels. We identify two *egl-19* gain-of-function mutations that are equivalent to the Timothy syndrome G402R and G406R mutations. We find that each of these *egl-19* gain-of-function mutations cause overextension of the PLM axon, leading to the misplacement of electrical synapses. Moreover, we find that *egl-19 gain-of-function* mutations interact genetically with a homolog of the autism-associated *WDFY3* selective autophagy gene to disrupt axon development and alter behavior.

## Results

### A VGCC mutation that causes autism in humans and disrupts axon termination in *C*. *elegans*

**Fig 1:**
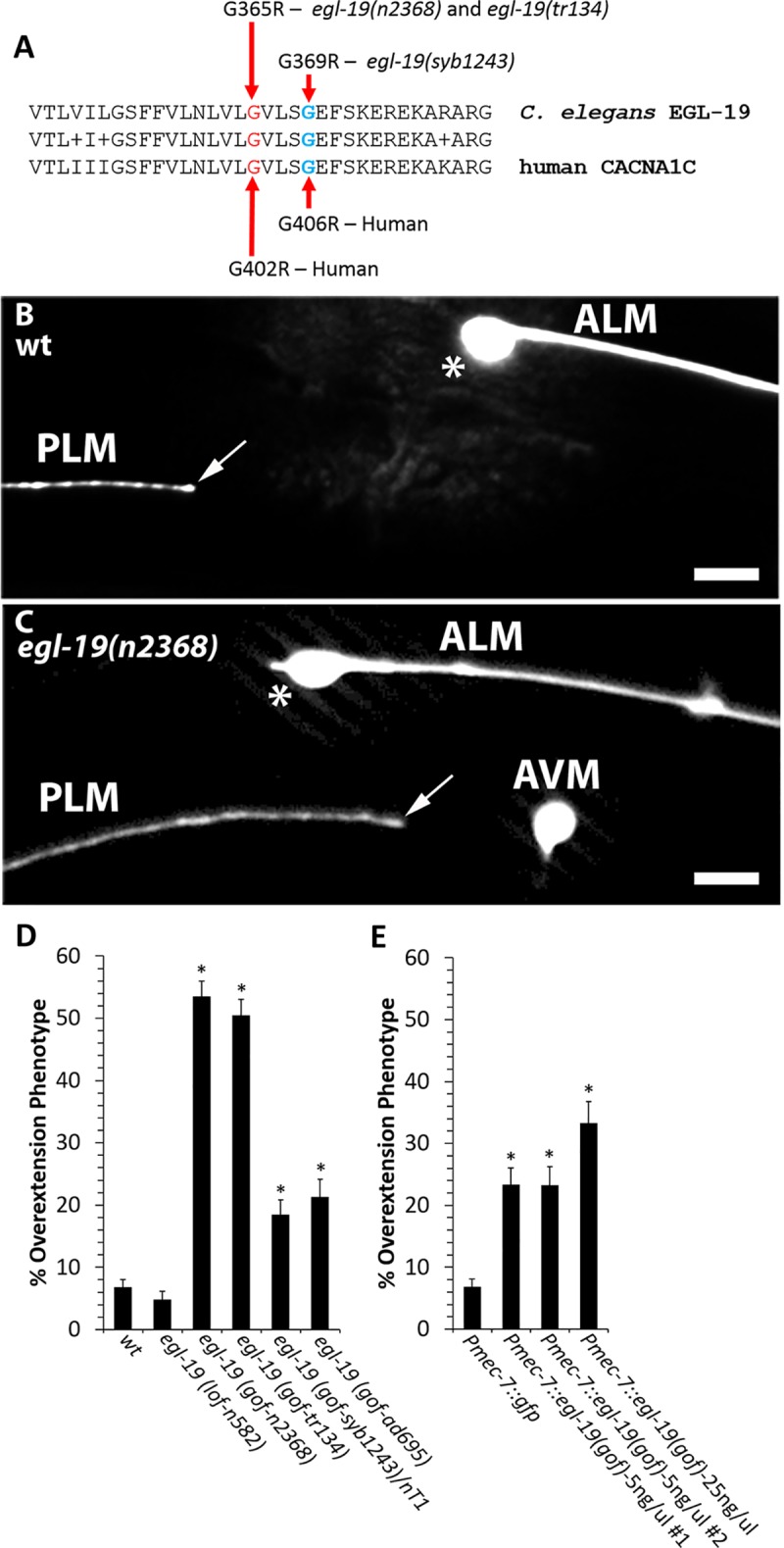
Mutations equivalent to the G402R and G406R Timothy syndrome mutations cause PLM axon termination defects. **(A)** The *egl-19(n2368)* and *egl-19(tr134)* mutations are equivalent to the Timothy Syndrome mutation in *CACNA1C* that causes autism in humans. The *egl-19(syb1243)* mutation is equivalent to the CACNA1C G406R mutation that causes Timothy Syndrome in humans. **(B)** Example of normal axon termination in a L4 stage wild-type PLM neuron, where the axon terminates posterior to the ALM cell body. **(C)** Example of axon termination defect in a L4 stage *egl-19(gof)* mutant, where the axon terminates anterior to the ALM cell body. Axons are visualized with the *muIs32* transgene that encodes *Pmec-7*::*gfp*. Arrows point to the tip of the PLM axon. Asterisk marks the ALM cell body. Scales bar are 10um. **(D)** Gain-of-function mutations in *egl-19* cause axon termination defects. The *egl-19(lof)* mutation does not affect axon termination. **(E)** Transgenic expression of EGL-19(GOF) specifically within touch receptor neurons causes axon termination defects. The *Pmec-7*::*egl-19(gof)* transgenes use the *mec-7* promoter to drive expression of an *egl-19* cDNA that includes a mutation identical to the *egl-19(n2368)* mutation. For *Pmec-7*::*egl-19(gof)*-5 ng/ul, 2 independent transgenic strains were analyzed. For *Pmec-7*::*egl-19(gof)*-25 ng/ul, 1 transgenic strain was analyzed. Between 200 and 400 axons were observed in L4 stage hermaphrodites per genotype. Asterisks indicate statistically significant difference, Z-test for proportions (***p<0.0001). Error bars represent the standard error of the proportion.

Both common and rare variants in the human *CACNA1C* gene have been associated with autism and other neurodevelopmental disorders [1, 2, 8, 9, 12, 24–26]. The Timothy syndrome mutations, G402R, G402S and G406R in human CACNA1C, are of particular interest because each causes autism with high penetrance [10, 27, 28]. To establish a model for investigating how the Timothy syndrome mutations affect neurodevelopment, we searched for an equivalent mutation in *egl-19*, the *C*. *elegans* orthologue of *CACNA1C*. We found that the *egl-19(n2368)* mutation (hereafter referred to as *egl-19(gof))* encodes a G365R variant of EGL-19 that is equivalent to the G402R Timothy syndrome variant in human *CACNA1C* (Fig 1A). In both human CACNA1C and *C*. *elegans* EGL-19, this Timothy syndrome mutation is a gain-of-function variant: it disrupts slow inactivation of the voltage-gated channel, thereby increasing calcium permeation [10].

To determine how the Timothy syndrome mutation affects neurodevelopment, we observed the PLM touch receptor neuron in *egl-19(gof)* mutants. The PLM cell body is located in the tail, with an axon extending along the lateral body wall. For this experiment we used two independently isolated *egl-19(gof)* alleles, *egl-19(n2368)* and *egl-19(tr134)*. Both of these alleles produce a G365R mutation in EGL-19, which is equivalent to the G402R Timothy syndrome mutation in humans (Fig 1A) [29, 30]. In wild-type animals, nearly all of the PLM axons terminate posterior to the ALM cell body (Fig 1B and 1D). However, in the *egl-19(n2368)* and *egl-19(tr134)* G365R gain-of-function mutants, around 52% of the PLM axons terminate anterior to the ALM cell body (Fig 1C and 1D). By contrast, *egl-19(lof)* mutants exhibit normal PLM axon termination (Fig 1D), with nearly all PLM axons terminating posterior to the ALM cell body. We also tested the *egl-19(ad695)* gain-of function mutation, which has previously been characterized as a weaker gain-of-function relative to *egl-19(n2368)* [30]. Consistent with these prior observations, we found that the *egl-19(ad695)* gain-of-function mutation causes axon termination defects with a lower penetrance relative to *egl-19(n2368)* and *egl-19(tr134)* (Fig 1D).

In humans, Timothy syndrome is also caused by a G406R mutation in *CACNA1C* [10]. Therefore, we tested *egl-19(syb1243)*, *a mutation that produces a G369R* mutation in EGL-19, which *is equivalent to* G406R in human CACNA1C (Fig 1A). We found that the *egl-19(syb1243)* mutation is homozygous lethal, with no maternal rescue. However, we observed the PLM axon in *egl-19(syb1243)* heterozygotes and found that around 18% of the PLM axons had axon termination defects (Fig 1D). Together, these observations indicate that mutations equivalent to the Timothy syndrome mutations can cause defects in axon targeting.

To determine if the Timothy syndrome mutation functions cell autonomously to disrupt axon termination, we used *Pmec-7*::*egl-19(gof)* transgenes to express the EGL-19 G365R mutant protein specifically within touch receptor neurons (PLM, ALM, AVM and PVM) (Fig 1E). We tested one transgene that was created by injecting *Pmec-7*::*egl-19(gof)* at a concentration of 5 ng/ul and another at 25 ng/ul. We found that both of these transgenes caused axon termination defects. These results suggest that EGL-19(GOF) functions cell autonomously in the PLM neuron to disrupt axon termination.

### Wild-type EGL-19 and other VGCC subunits negatively regulate axon termination

To determine if and how wild-type EGL-19 regulates axon termination, we conducted genetic analysis with mutations in the genes that encode the RPM-1 (PAM, Highwire) signaling pathway. RPM-1 is an E3 ubiquitin ligase that promotes axon termination by ubiquitinating DLK-1 (MAP3K12), thereby marking it for proteasomal degradation [31–33]. This function of RPM-1 is mediated through an interaction between RPM-1 and a SCF (Skp/Cullin/F-box) complex that includes the FSN-1 (FBXO45) F-box protein. In addition, RPM-1 also promotes axon termination by functioning with GLO-4 (RCBTB1), a guanine nucleotide exchange factor (GEF) for the GLO-1 (RAB38) Rab GTPase [34]. GLO-4 functions with RPM-1, but in parallel to FSN-1, to promote axon termination.

**Fig 2:**
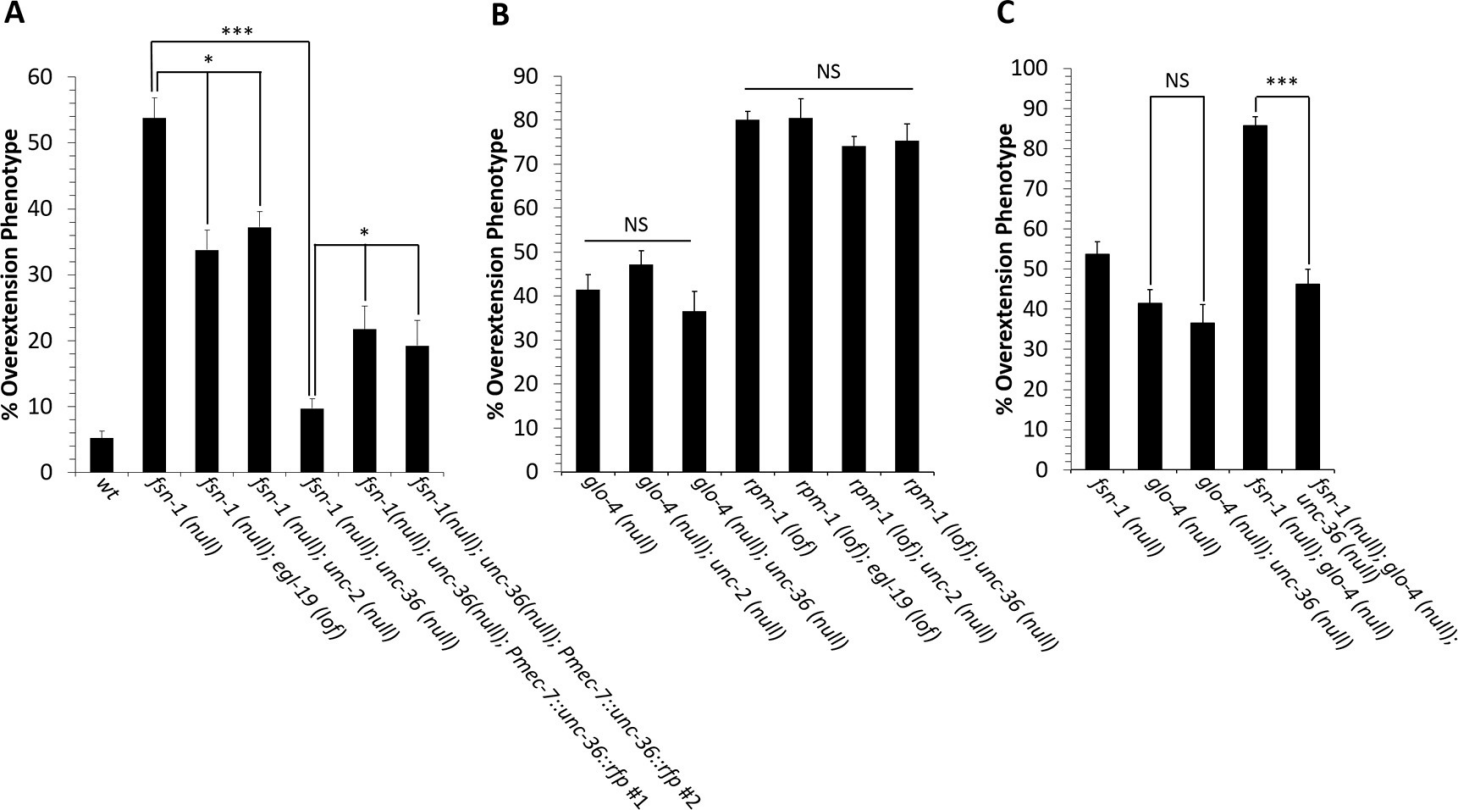
VGCC genes negatively regulate axon termination. **(A)** Loss-of-function mutations in VGCC genes suppress axon termination defects caused by the *fsn-1(null)* mutation. The *egl-19* gene encodes the pore-forming subunit of the L-type VGCC. The *unc-2* gene encodes the pore-forming subunit of the P/Q-type VGCC and the *unc-36* gene encodes the Alpha2-Delta3 subunit that works with both the L-type and P/Q-type VGCCs. **(B)** Mutations in VGCC genes do not suppress axon termination defects caused by loss-of-function mutations in *glo-4* or *rpm-1*. **(C)** Loss of VGCC function can partially suppress axon termination defects caused by the *fsn-1(null)*; *glo-4(null)* double mutant background. Asterisks indicate statistically significant difference, Z-test for proportions (*p<0.005, ***p<0.0001). Between 200 and 400 axons were observed in L4 stage hermaphrodites per genotype using the *muIs32* transgene. Error bars represent the standard error of the proportion. For *Pmec-7*::*unc-36*::*rfp*, 2 independent transgenic strains were analyzed. Alleles: *fsn-1(null)* is *fsn-1(gk429)*, *egl-19(lof)* is *egl-19(n582)*, *unc-2(null)* is *unc-2(e55)*, *unc-36(null)* is *unc-36(e251)*, *glo-4(null)* is *glo-4(ok362)*, *rpm-1(lof)* is *rpm-1(ok364)*.

Since the *egl-19(gof)* mutation disrupts axon termination, it is possible that wild-type EGL-19 also negatively regulates axon termination. Alternatively, it is possible that *egl-19(gof)* acts in a neomorphic role not normally controlled by wild-type EGL-19. To determine the function of wild-type EGL-19, we conducted genetic analysis with a loss-of-function allele of *egl-19*. For this experiment, we used a null allele of the *fsn-1* that causes axon termination defects [34, 35]. In *fsn-1(null)* mutants, 53% of the PLM axons are overextended (Fig 2A). However, in *fsn-1(null)*; *egl-19(lof)* double mutants, the phenotype is suppressed to only 33% of PLM axons overextended. These observations suggest that wild-type EGL-19 acts to negatively regulate axon termination.

EGL-19 is the pore forming subunit of the L-type VGCC. To further explore the role of voltage-gated calcium channels, we tested loss-of-function mutations in other genes that encode VGCC components. The *unc-2* gene encodes the pore-forming subunit of the P/Q-type VGCC [36]. The human homolog of *unc-2*, CACNA1A, has also been associated with autism [1, 37, 38]. We found that a null allele of *unc-2* could also suppress axon termination defects caused by a *fsn-1(null)* mutation (Fig 2A). We also tested a null mutation in *unc-36*, which encodes the alpha2-delta3 subunit that works with both the EGL-19 and UNC-2 pore-forming subunits to modulate voltage dependence, activation kinetics, and calcium conductance [39–41]. The human homolog of UNC-36, CACNA2D3 has also been associated with autism [6, 8, 42]. We found that a null allele of *unc-36* could also suppress axon termination defects caused by a *fsn-1(null)* mutation (Fig 2A). Together, these observations suggest that both L-type and P/Q-type voltage-gated calcium channels can negatively regulate axon termination. We note that the different levels of suppression observed between *unc-36(null)*, *unc-2(null)* and *egl-19(lof)* mutations are likely the result of differing roles played by each VGCC subunit or could reflect different strengths of the alleles that we used.

To determine if VGCCs function cell-autonomously to regulate axon termination, we constructed a *Pmec-7*::*unc-36*::*rfp* transgene, which uses the *mec-7* promoter to drive expression of UNC-36::RFP within the touch receptor neurons. If UNC-36 functions cell autonomously, we expect that the *Pmec-7*::*unc-36*::*rfp* transgene will reverse the suppression of axon termination defects observed in *fsn-1(null)*;*unc-36(null)* double mutants relative to *fsn-1(null)* single mutants. Indeed, we found that *fsn-1(null)*;*unc-36(null)* double mutants with the *Pmec-7*::*unc-36*::*rfp* transgene had a higher penetrance of axon termination defects relative to *fsn-1(null)*;*unc-36(null)* double mutants without the *Pmec-7*::*unc-36*::*rfp* transgene (Fig 2A). These observations suggest that UNC-36 functions cell-autonomously to negatively regulate axon termination.

### VGCC regulation of axon termination is specific to the FSN-1 pathway

FSN-1 functions in parallel to GLO-4 to promote PLM axon termination [34, 43]. Although both pathways promote axon termination, they do so through distinct molecular mechanisms. Whereas FSN-1 is an F-box protein that regulates a MAP Kinase cascade [35], GLO-4 is a guanine nucleotide exchange factor for the GLO-1 Rab GTPase [43]. To determine if VGCCs regulate the GLO-4 pathway, we constructed *glo-4(null)*;*unc-2(null)* and *glo-4(null)*;*unc-36(null)* double mutants. We found that neither loss of *unc-2* nor loss of *unc-36* function suppresses the axon termination defects caused by loss of *glo-4* function, suggesting that VGCCs do not regulate the GLO-4 pathway (Fig 2B). To further explore the role of VGCCs within the context of the parallel FSN-1 and GLO-4 pathways, we constructed an *fsn-1(null)*;*glo-4(null)*;*unc-36(null)* triple mutant and a *fsn-1(null)*;*glo-4(null)* double mutant (Fig 2C). Consistent with prior studies, we found that *fsn-1(null)*;*glo-4(null)* double mutants had termination defects in 86% of PLM axons [34]. In the triple mutant, loss of *unc-36* function reduced this penetrance to 46%, which is similar to the *glo-4* single mutants. Together, these observations suggest that VGCCs can negatively regulate axon termination in response to the FSN-1 pathway, but not GLO-4 pathway.

The RPM-1 ubiquitin ligase functions with the FSN-1 F-box protein to negatively regulate downstream proteins [34, 35]. Loss-of-function mutations in these downstream proteins suppress the phenotype of loss-of-function mutations in FSN-1 and RPM-1. For example, FSN-1 and RPM-1 function together to negatively regulate the DLK-1 MAP Kinase [31]. Loss of DLK-1 function suppresses the phenotype that is caused by either loss of RPM-1 function or loss of FSN-1 function. Since loss of VGCC function can suppress the axon termination phenotype caused by loss of FSN-1 function, we considered the possibility that FSN-1 might function with RPM-1 to negatively regulate VGCCs. If this is true, loss of VGCC function should suppress the axon termination defect caused by loss of RPM-1 function. However, we found that axon termination defects caused by loss of RPM-1 function could not be suppressed by loss of function mutations in *unc-36*, *unc-2* or *egl-19* (Fig 2B). These observations suggest that VGCCs are not downstream targets of RPM-1 and FSN-1.

### The *egl-19(gof)* mutation alters PLM axon connectivity

**Fig 3:**
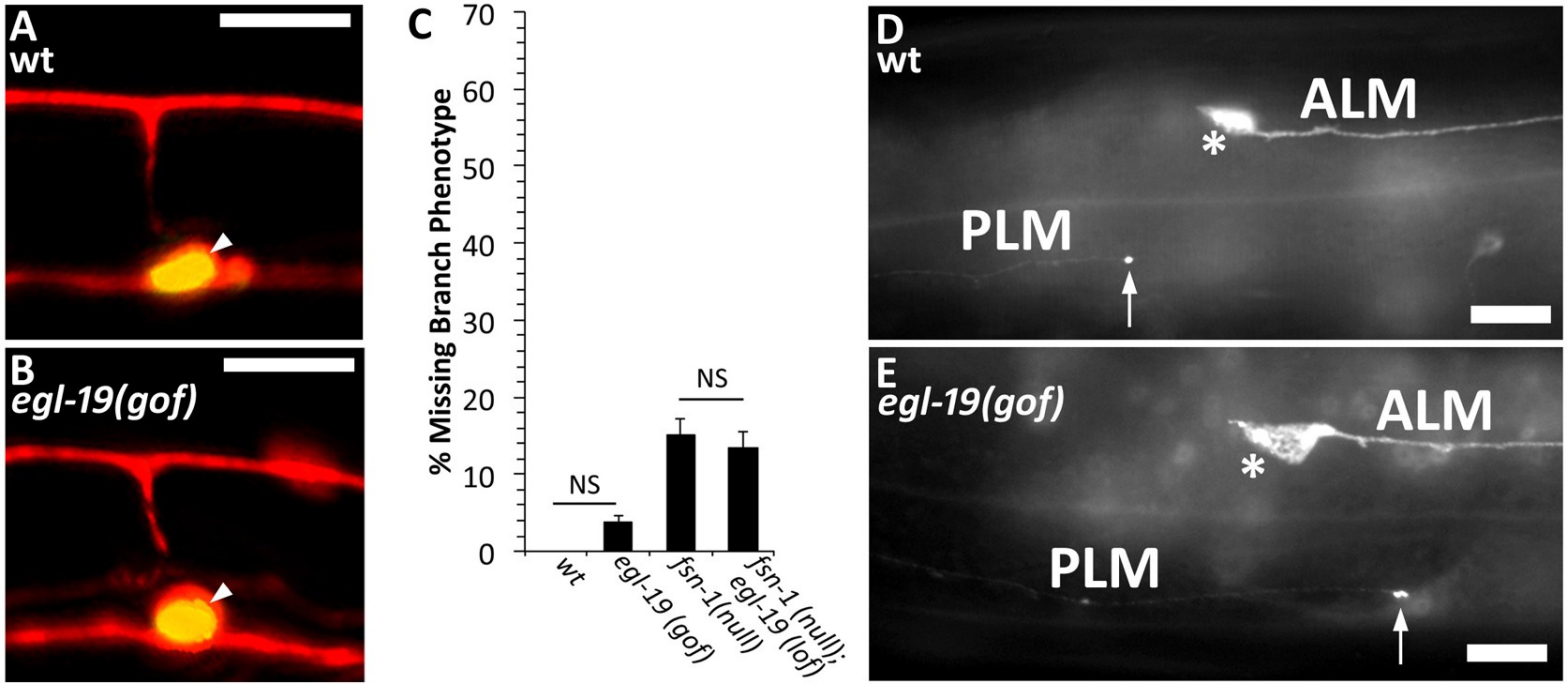
The *egl-19(gof)* Timothy syndrome mutation causes mislocalization of PLM electrical synapses. **(A)** Example of normal PLM chemical synapse in a wild-type animal. The PLM axon and branch are labeled in red. **(B)** Example of chemical synapse in a *egl-19(gof)* mutant. In A and B, the axon and its synaptic branch are shown in red as visualized with the *jsls973* transgene that encodes *Pmec-7*::*rfp*. The chemical synapse is shown in green as visualized by the *jsls821* transgene encodes *Pmec-7*::*gfp*::*rab-3*. Arrowheads mark the synaptic vesicles, which appear yellow due to overlap with the red signal. **(C)** The PLM synaptic branch is unaffected by changes in *egl-19* function. The *egl-19(gof)* mutation does not cause defects in the PLM synaptic branch. The *egl-19(lof)* mutation does not suppress the PLM synaptic branch defect caused by the *fsn-1(null)* mutation. Between 200 and 400 synaptic branches were observed in L4 stage hermaphrodites per genotype using the *muIs32* transgene. Error bars represent the standard error of the proportion. **(D)** Example of zone 2 electrical synapses at the tip of the PLM axon in wildtype animals. **(E)** Example of zone 2 electrical synapses at the tip of the PLM axon in *egl-19(gof)* mutants. The *egl-19(gof)* mutation causes mislocalization of PLM zone 2 electrical synapses. The zone 2 electrical synapses should be localized posterior to the ALM cell body. However, in *egl-19(gof)* mutants, the zone 2 electrical synapses are aberrantly localized anterior to the ALM cell body. Electrical synapses were visualized with the *yadIs12* transgene that encodes *Pmec-4::gfp::unc-9 [46].* Arrow marks electrical synapses at the tip of the PLM axon. Asterisk marks the ALM cell body. Scalebars are 10um. Alleles: *egl-19(gof)* is *egl-19(n2368)*, *fsn-1(null)* is *fsn-1(gk429)*, *egl-19(lof)* is *egl-19(n582)*.

To determine if the *egl-19(gof)* mutation affects connectivity of the PLM axon, we examined its chemical and electrical synapses. In wild-type animals, the PLM axon extends a synaptic branch that forms a cluster of chemical synapses onto axons in the ventral nerve cord [32]. We used a *mec-7*::*rfp* transgene [44] to visualize the PLM synaptic branch and found that it appears normal in *egl-19(gof)* mutants (Fig 3A and 3B). We also used a *mec-7*::*gfp*::*rab-3* transgene [45] to visualize synaptic vesicles and found that these also appear normal in *egl-19(gof)* mutants (Fig 3A and 3B). We measured the length of the synaptic vesicle clusters in wildtype and *egl-19(gof)* mutants and found no significant difference (t-test, p>0.05): *egl-19(gof)* = 4.7±0.11μm (n = 50); wildtype = 5.09±0.18μm (n = 50). Moreover, consistent with prior findings [34], we found that about 15% of *fsn-1* null mutants were missing the PLM branch (Fig 3C). However, this missing branch phenotype was not suppressed in *fsn-1(null)*; *egl-19(lof)* double mutants (Fig 3C). These observations suggest that the EGL-19(GOF) mutation does not affect the PLM synaptic branch or its chemical synapses. Moreover, wildtype EGL-19 does not affect the PLM branch. However, we cannot rule out the possibility that the synaptic branch and chemical synapses are affected in more subtle ways.

We next asked if the *egl-19(gof)* mutation affects electrical synapses. In wild-type PLM axons, electrical synapses are clustered in two distinct zones [46]. Zone 1 electrical synapses are located close to the PLM cell body, whereas zone 2 electrical synapses are located at the PLM axon tip, which is posterior to the ALM cell body (Fig 3D). Since the *egl-19(gof)* mutation causes overextension of the PLM axon, it is possible that it could also cause misplacement of zone 2 electrical synapses to a location anterior to the ALM cell body. Alternatively, it is possible that *egl-19(gof)* causes axon overextension, but leaves the zone 2 synapses in their normal location posterior to the ALM cell body. To differentiate between these two possibilities, we used a *mec-7*::*unc-9*::*gfp* transgene [46] to visualize the UNC-9 Innexin, a marker for electrical synapses [46]. We found that the *egl-19(gof)* mutation caused misplacement of the zone 2 electrical synapses to a point anterior to the ALM cell body (Fig 3E). Misplacement of the electrical synapse occurred in 52.9±7.0% of *egl-19(gof)* PLM axons, but only in 7.1±3.4% of wildtype PLM axons (n = 50 for both genotypes, significantly different by z-test for proportions, p<0.0001). Therefore, the PLM axon overextension caused by *egl-19(gof)* is also associated with misplacement of zone 2 electrical synapses.

### The *egl-19(gof)* mutation interacts with selective autophagy genes to disrupt axon termination

**Fig 4:**
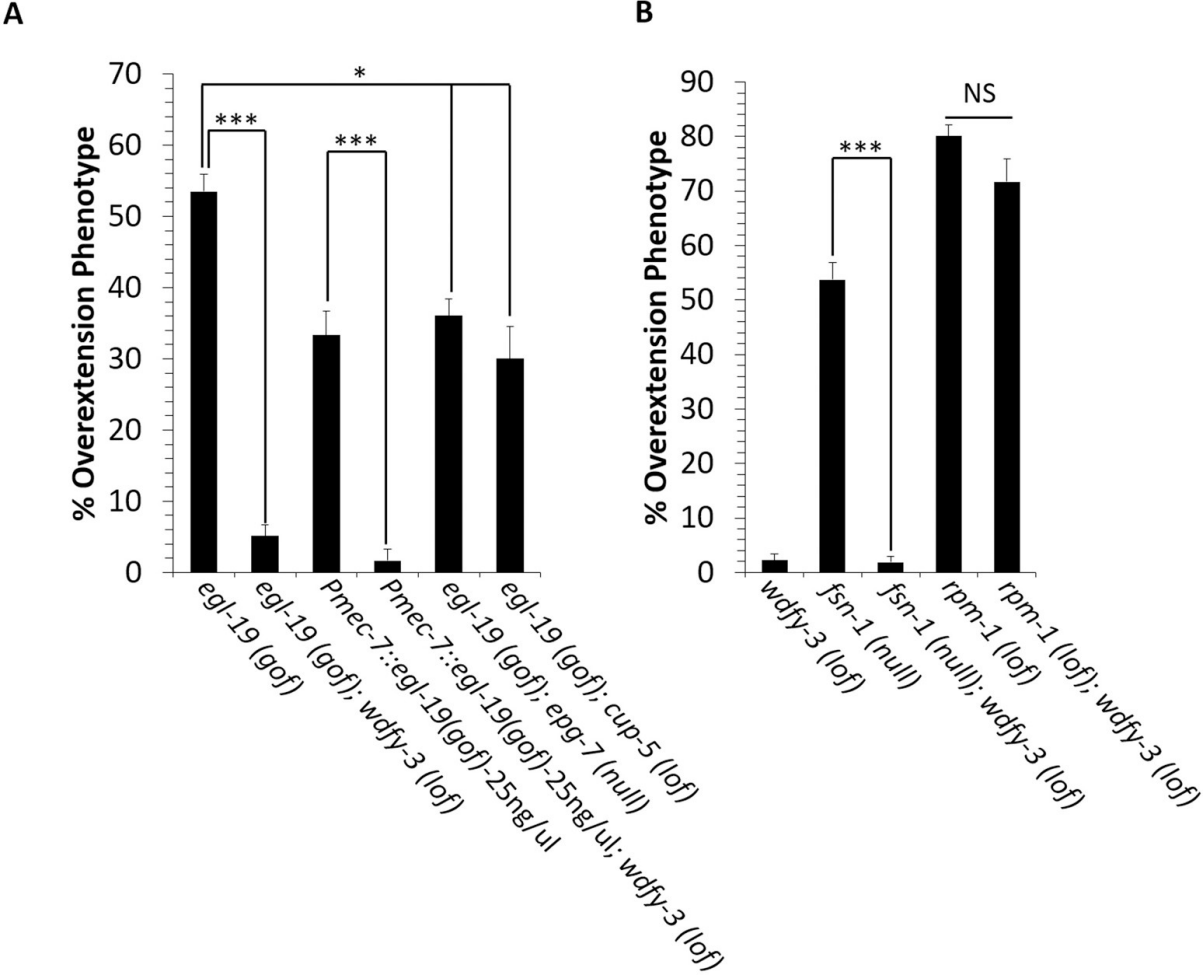
EGL-19(GOF) functions with selective autophagy to cause axon termination defects. **(A)** Axon termination defects caused by the *egl-19(gof)* mutation are suppressed by loss-of-function mutations in *wdfy-3(lof)*, *epg-7(null)* and *cup-5(lof)*. **(B)** Axon termination defects caused by the *fsn-1(null)* mutation are suppressed by the *wdfy-3(lof)* mutation. However, axon termination defects caused by *rpm-1(lof)* mutation are not suppressed by the *wdfy-3(lof)* mutation. Axons are visualized with the *muIs32* transgene that encodes *Pmec-7*::*gfp*. Asterisks indicate statistically significant difference, Z-test for proportions (*p<0.005, ***p<0.0001). Error bars represent the standard error of the proportion. n = 200–400 axons per genotype. Alleles: *egl-19(gof)* is *egl-19(n2368)*, *wdfy-3(lof)* is *wdfy-3(ok912)*, *epg-7(null)* is *epg-7(tm2508)*, *cup-5(lof)* is *cup-5(ar465)*, *rpm-1(lof)* is *rpm-1(ok364)*, *fsn-1(null)* is *fsn-1(gk429)*.

As part of an ongoing effort to test autism-associated genes for roles in axon development, we identified a genetic interaction between *egl-19(gof)* and *wdfy-3*, a homolog of the autism-associated *WDFY3* selective autophagy gene. For this experiment, we used the *wdfy-3(ok912)* deletion allele, hereafter called *wdfy-3(lof)*. This allele is likely to be a null or strong loss of function because it creates a frameshift that disrupts the FYVE domain, WD repeat domain and nearly all of the beach domain. We found that the PLM axon was normal in *wdfy-3(lof)* single mutants. In *egl-19(gof)*; *wdfy-3(lof)* double mutants, the axon termination defects observed in *egl-19(gof)* single mutants were almost completely suppressed (Fig 4A). Moreover, PLM axon termination defects caused by transgenic expression of EGL-19(GOF) in touch receptor neurons were also suppressed by *wdfy-3(lof)*. The PLM neurons are likely to express WDFY-3 because RNAseq on purified touch receptor neurons identified *wdfy-3* mRNA transcripts [47]. The *wdfy-3* gene is an orthologue of the human autism-associated *WDFY3* gene that encodes a protein required for cargo selection during selective autophagy [48, 49]. Therefore, these observations establish a genetic pathway between two autism-associated genes that regulates axon termination.

To further explore a potential interaction between selective autophagy and EGL-19(GOF), we constructed double mutants between *egl-19(gof)* and mutations in two other genes that are expected to disrupt selective autophagy: *epg-7* (RB1CC1, FIP200) and *cup-5 (Mucolipin-3)*. The *epg-7* gene encodes an additional component required for selection of cargo for autophagy [50], and the *cup-5* gene encodes a scaffold protein that promotes lysosome biogenesis [51, 52]. We found that axon termination defects caused by the *egl-19(gof)* mutation could be suppressed by either a likely null mutation in *epg-7* or a hypomorphic mutation in *cup-5* (Fig 4A). These observations suggest that the *egl-19(gof)* mutation causes axon termination through a mechanism that requires selective autophagy.

### WDFY-3 negatively regulates PLM axon termination

Having found that *wdfy-3* can negatively regulate axon termination in the *egl-19(gof)* mutant, we wanted to ask if *wdfy-3* could also regulate axon termination independently of the gain-of-function *egl-19* mutation. For this experiment, we used a *fsn-1(null)* mutation to induce axon termination defects. We found that loss of *wdfy-3* function completely suppresses the axon termination defects caused by the *fsn-1(null)* mutation (Fig 4B). Like the VGCC loss-of-function mutants, we also found that the *wdfy-3(lof)* mutant does not suppress axon termination defects caused by *rpm-1(lof)*. Together, these observations suggest that WDFY-3, like VGCCs, can regulate axon termination signaling downstream of FSN-1, but is not a downstream target of FSN-1 and RPM-1.

### The *egl-19(gof)* mutation interacts with the *wdfy-3* selective autophagy gene to regulate habituation to light touch

The PLM neuron is a mechanosensory neuron that is responsible for sensing light touch in the posterior of *C*. *elegans* [53]. When light touch is applied to the tail, the animal responds by moving forward [54–56]. However, after repeated touches, the animal habituates and becomes less likely to respond to each touch. Since we observed that the *egl-19(gof)* mutation alters the morphology of the PLM neuron, we wanted to determine if this mutation also alters the response to light touch.

**Fig 5:**
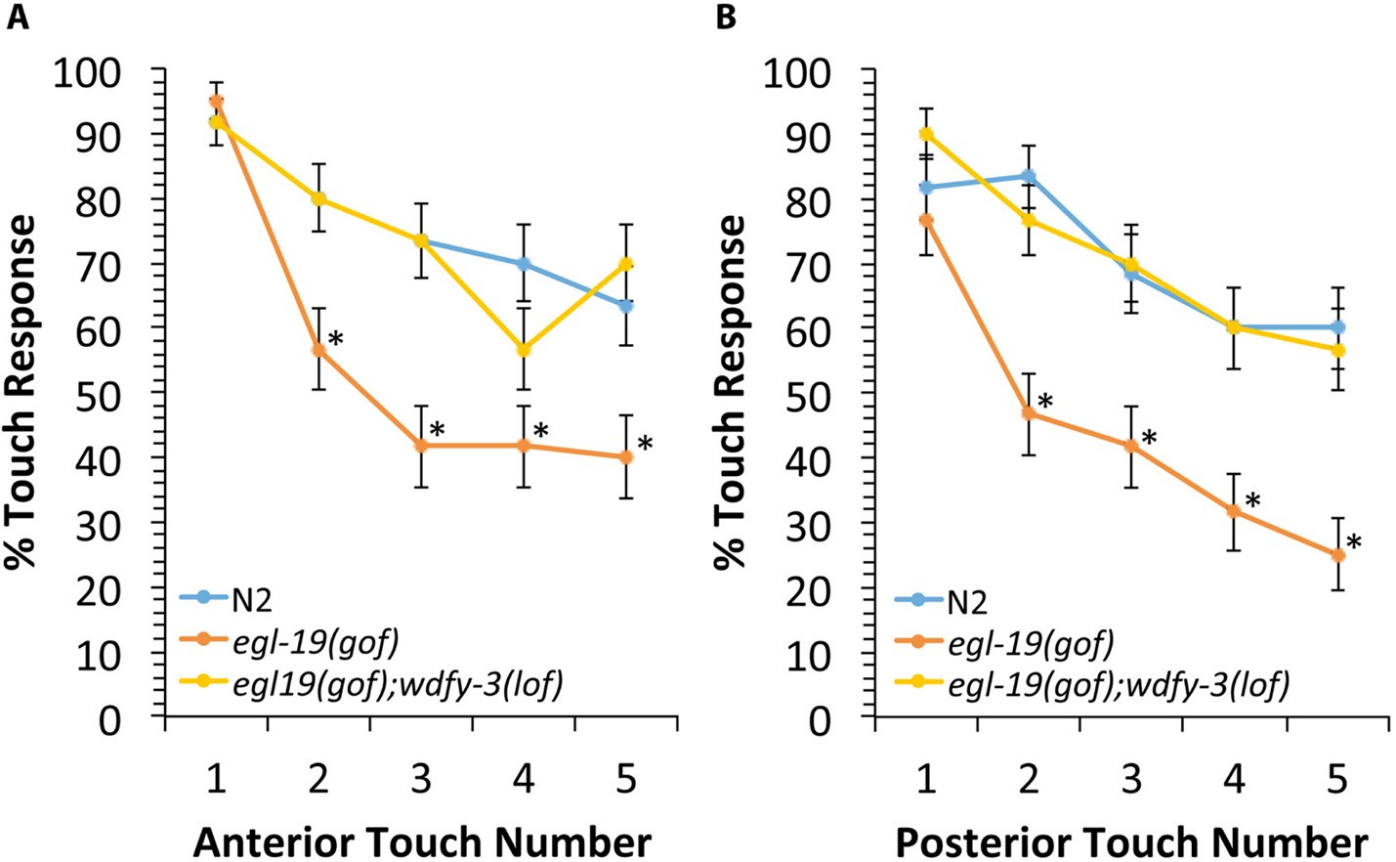
The *wdfy-3* gene functions with the *egl-19(gof)* mutation to alter habituation to light touch. Animals were subjected to eyelash touches alternating between the head and tail and the response rate was recorded after each touch. **(A)** Response rate for anterior touches. **(B)** Response rate for posterior touches. For both anterior and posterior touches habituation was significantly increased in *egl-19(gof)* mutants relative to wild-type. This change in habituation in *egl-19(gof)* mutants was suppressed by *wdfy-3(lof)*. Asterisks indicate statistically significant difference compared to wild type worms, Z-test for proportions (*p<0.0001). Error bars represent the standard error of the proportion. For each genotype, the assay was repeated 3 times by 3 different observers who were blind to the genotype. Each experiment included 20 worms for a total n of 60 for each genotype. Alleles: *egl-19(gof)* is *egl-19(n2368)*, *wdfy-3(lof)* is *wdfy-3(ok912)*.

We conducted a touch assay to determine if the *egl-19(gof)* mutation affects the response to light touch. Each animal was subjected to ten eyelash touches alternating between the head and tail (Fig 5). For the initial touch, there was no significant difference in the response rate between *egl-19(gof)* mutants and wild-type animals. However, for each subsequent touch, the *egl-19(gof)* mutants had a significantly lower response rate relative to wild-type animals. These observations suggest that the *egl-19(gof)* mutation enhances habituation to light touch.

Because we found that the *egl-19(gof)* mutation interacts with *wdfy-3* to disrupt axon termination, we next asked if this genetic interaction can also affect habituation to light touch. If the *egl-19(gof)* mutation functions with *wdfy-3* to alter habituation, we expect that loss of *wdfy-3* function will reduce the effect of the *egl-19(gof)* mutation on habituation. Indeed, we found that in *egl-19(gof);wdfy-3(lof)* mutants, the habituation to light touch was not significantly different relative to wild-type animals. Together, these observations suggest that the *egl-19(gof)* mutation acts through *wdfy-3* to affect both axon termination and habituation to light touch.

## Discussion

The Timothy syndrome mutation in *CACNA1C* has the unusual property of being causative for autism with high penetrance, providing an opportunity to discover the downstream cellular processes that are perturbed to cause autism. However, the cellular processes that interact with this mutation to give rise to autism have remained unknown. To address this question, we created a disease model in *C*. *elegans* that utilizes a mutation equivalent to the Timothy syndrome mutation in humans. Our results reveal that selective autophagy genes interact with the Timothy syndrome mutation to disrupt axon termination and alter behavior. Because common variants of *CACNA1C* are associated with autism, it is likely that this mechanism will be broadly applicable to autism in humans.

### An understanding of genetic interactions between variants is key to understanding autism

Only a small fraction of autism cases are thought to be caused by a single variant. Rather, most cases of autism are thought to be caused by genetic interactions between variants [9, 14, 57]. For example, likely gene-disrupting (LGD) mutations have been associated with 15–20% of autism cases. In addition, autistic individuals carry more missense mutations in autism-associated genes relative to healthy controls [9, 58].

However, both LGD and missense mutations are rare and therefore almost always heterozygous. Therefore, in most cases, it is thought that each of these mutations have little or no effect on their own. Thus, it is likely that the disorder arises from genetic interactions between autism-associated variants. Indeed, statistical analysis of sequencing data suggests that autism arises from the combined action of multiple variants [9, 14, 59].

Although the heritability of autism has been estimated at 83% [60], the complexity of the genetic interactions that give rise to autism make it difficult to predict and diagnose autism from whole genome sequencing data. In fact, with current knowledge, no genetic cause can be found from whole genome sequencing data for most cases of autism. The solution to this challenge could come from genetic analysis. For example, in most cases, single heterozygous null mutations have no phenotype. However, many cases exist to show that an animal carrying two heterozygous null mutations can exhibit a phenotype when each of the mutated genes function in the same genetic pathway [61, 62]. Thus, if an individual is heterozygous for two LGD variants in each of two autism-associated genes that function in a pathway, this individual would carry a higher risk for autism. Therefore, knowledge of the pathways that link autism-associated genes will help promote our understanding of the genetic basis of autism.

### Selective autophagy functions with EGL-19(GOF) to alter axon development and behavior

A key finding of our study is the identification of a genetic interaction between the homologs of two autism-associated genes, *egl-19* and *wdfy-3*. Our genetic analysis indicates that *wdfy-3* and other selective autophagy genes are required for the *egl-19(gof)* mutation to disrupt axon termination. Moreover, we also find that *wdfy-3* can negatively regulate axon termination independently of the *egl-19(gof)* mutation. These genetic interactions could be explained by a few different molecular models. First, it is possible that EGL-19 functions in a pathway with WDFY-3 to negatively regulate axon termination. Alternatively, it is possible that EGL-19 and WDFY-3 function in two separate parallel pathways that can both negatively regulate axon termination. We favor the former model, because both *egl-19(lof)* and *wdfy-3(lof)* are able to suppress axon termination defects caused by loss of *fsn-1* function, but not by loss of *rpm-1* function. Moreover, the strength of the suppression of the *egl-19(gof)* phenotype by the *wdfy-3(null)* mutation is most consistent with the idea that EGL-19 and WDFY-3 function together in a pathway. If EGL-19 and WDFY-3 do function together in a pathway, it is possible that the Timothy syndrome mutation induces excessive selective autophagy that causes a disruption of axon termination. As an alternative, it is also possible that selective autophagy could function upstream of the EGL-19(GOF) mutant protein. In this scenario, selective autophagy could promote the function of the EGL-19(GOF) protein by affecting its turnover, stability or localization.

The genetic interaction between *egl-19* and *wdfy-3* also regulates habituation to light touch. It is possible that this genetic interaction affects behavior by functioning in the developing nervous system to regulate connectivity. Alternatively, it is possible that the genetic interaction between *egl-19* and *wdfy-3* functions in the mature nervous system to regulate neural function. Although our data cannot distinguish between these two possibilities, recent work on mice favor the former possibility [63]. Conditional knockout of *CACNA1C* in forebrain neurons during development results in anxiety in adult mice, whereas knockout of *CACNA1C* during adulthood does not. These observations lend support to the possibility that CACNA1C acts during development to alter circuit formation, which in turn affects behavior in the adult.

The interaction between the Timothy syndrome mutation and *wdfy-3* provides biological evidence for a role of selective autophagy in autism. A major challenge in autism genetics is to confirm and characterize the roles of autism candidate genes. For example, *WDFY3* is a candidate gene for autism because whole genome sequencing has found that 3 out of 6707 sequenced autism genomes contain a heterozygous *de novo* likely-gene-disrupting mutation in *WDFY3* [6, 8, 42, 64]. However, despite this association, it is not possible to determine if *WDFY3* variants contribute to the cause of autism. Our results place genes that promote selective autophagy in a pathway with a mutation that is causative for autism in humans, thereby providing the first biological evidence for a role of selective autophagy in autism.

Selective autophagy is also required for normal axon development. Aside from its function with EGL-19(GOF) in inducing axon defects, our results suggest that WDFY-3 also functions independently of EGL-19(GOF) to regulate axon development. Consistent with this idea, loss of *WDFY3* causes the disorganization and loss of many commissural axon tracts in mice [65]. Loss of *WDFY3* in mice also attenuates the response to guidance cues *in vitro*, suggesting that selective autophagy could regulate the response to guidance cues. Despite these insights, the mechanism through which selective autophagy regulates axon targeting is currently unknown. Based on our genetic analysis, we propose a mechanism whereby selective autophagy functions with voltage-gated calcium channels to regulate the response to axon targeting cues.

Selective autophagy and bulk autophagy may have distinct functions in neurodevelopment. Whereas our results suggest a mechanism for selective autophagy in the negative regulation of axon termination, prior studies have reported a role for bulk autophagy in promoting synapse development and inhibiting axon growth. In cultured mouse neurons, knockdown of an autophagy gene promotes axon growth, whereas induction of autophagy inhibits axon growth [66]. In *Drosophila*, autophagy promotes development of the neuromuscular junction [67]. In *C*. *elegans*, autophagosomes form at synaptic sites and are required for presynaptic assembly [68, 69]. This role for autophagy in synaptogenesis is specific to bulk autophagy, since mutations in selective autophagy genes do not affect synaptogenesis [69]. Interestingly, our results suggest that selective autophagy may regulate axon termination without affecting synaptogenesis.

Selective autophagy could function with VGCCs to regulate other aspects of autism-related pathology. Whereas our study focuses on the role of the Timothy syndrome mutation in misregulating axon termination and behavior, previous work has found that the Timothy syndrome mutation can promote activity-dependent dendrite retraction in cultured mouse neurons and can inhibit the elaboration of mouse dendrites *in vivo* [70]. The downstream cellular mechanisms for this effect on dendrites are not yet known, but it is possible that selective autophagy could also be involved in this process. Alternatively, it is possible that the Timothy syndrome mutation functions through distinct mechanisms to affect dendrite development and axon development.

### Potential role for VGCCs in regulating the RPM-1 pathway

Our results identify specific genetic interactions between VGCC genes and RPM-1 pathway genes. These genetic interactions indicate that loss of VGCC function suppresses axon termination events that are caused by loss of *fsn-1* function, but not loss of *glo-4* function. Moreover, we find that loss of VGCC function does not suppress defects caused by loss of *rpm-1* function, but can partially suppress defects caused by the double loss of *fsn-1* and *glo-4*. Taken together, these observations suggest that VGCCs specifically regulate axon termination signaling downstream of FSN-1, but are not themselves downstream targets of RPM-1 and FSN-1.

The genetic interactions between the VGCC genes and RPM-1 pathway genes could be explained by a model where VGCCs negatively regulate an unknown protein that functions with RPM-1 to enhance signaling events that promote axon termination downstream of FSN-1, but not GLO-4 (S1 Fig). In fact, prior work has found that FSN-1 binds to RPM-1 and promotes axon termination by negatively regulating the DLK-1 MAP kinase signaling pathway [31, 35]. Moreover, the PPM-1 phosphatase also binds to RPM-1 and promotes axon termination by negatively regulating DLK-1 MAPK signaling [71]. Thus, it is possible that EGL-19 might repress axon termination by negatively regulating PPM-1, or another protein that plays a similar role (S2 Fig).

### Role for VGCC-mediated calcium transients in axon growth

Our study focuses on the genetic mechanisms that mediate the role of VGCC genes in axon termination, but does not address how alterations in calcium permeation might be involved in this process. Interestingly, a recent study of cultured prenatal mouse neurons has revealed that VGCCs function during axon outgrowth to produce calcium transients that have very different properties compared to those produced during synaptic transmission [72]. These transients have been named Spontaneous Regenerative Calcium Transients (SRCaTs) and are mediated by Ca_V_1.2, which includes the homolog of EGl-19, CACNA1C. Unlike its function in adult neurons, Ca_V_1.2 appears to open near resting potential, suggesting that the Ca_V_1.2 channel may open spontaneously in developing axons. Knockout of *CACNA1C* in these cultured neurons causes a decrease in axon growth. Thus, Ca_V_1.2 functions in axons to regulate axon growth, using a mechanism that is very different than how it functions in synaptic transmission. The role of SRCaTs in regulating axon growth are unknown. However, our results suggest the possibility that these SRCaTs may regulate signaling downstream of FSN-1 (FBXO45), utilizing a mechanism that involves selective autophagy.

### Potential role for common *CACNA1C* variants in affecting selective autophagy and axon development in autism

We propose that the effect of *CACNA1C* variation in altering axon development is not limited to the Timothy syndrome mutation, but rather extends to the other autism-associated *CACNA1C* variants. This idea is supported by our genetic analysis suggesting that the effect of the *egl-19(gof)* Timothy syndrome mutation on axon termination is not neomorphic, but rather reflects an increase in the normal function of *egl-19*. Therefore, other gain-of-function and loss-of-function variants in CACNA1C could contribute to autism by altering axon development. Consistent with this idea, statistical analysis has identified some candidate variants in VGCC genes that are likely to be gain-of-function and others that are likely to be loss-of-function [6, 10, 11, 42, 73]. Therefore, we speculate that both under-activation and over-activation of the signaling pathways that promote axon termination could contribute to autism.

The Timothy syndrome mutation is a very rare *de novo* mutation, and is therefore only responsible for a very tiny fraction of autism cases. However, several common variants in *CACNA1C* have also been associated with autism [1, 2, 74]. For example, the A genotype at the rs1006737 locus in *CACNA1C* confers risk for autism and is present in about 33% of the human population. This A genotype at rs1006737 is located within a large intron and is thought to cause *CACNA1C* gain-of-function because neurons with the risk genotype have higher levels of *CACNA1C* mRNA and increased L-type calcium currents relative to neurons with the non-risk genotype [75]. Therefore, this risk variant may be associated with a gain-of-function of *CACNA1C* that could disrupt axon development in a way analogous to the Timothy syndrome mutation. However, the small effect size of the rs1006737 locus suggests that this is a relatively weak *CACNA1C* gain-of-function.

Although common alleles have a small effect size relative to the Timothy syndrome mutation, they could interact with risk variants in other genes that function in a genetic pathway with *CACNA1*C. For example, the rs1006737 risk variant could provide a weak gain-of-function in *CACNA1C* that does not cause autism on its own. However, the rs1006737 risk variant could synergize with a gain-of-function risk variant in *WDFY3* to contribute to autism. Alternatively, a weak loss of function in *CACNA1C* could synergize with a weak loss-of-function in *WDFY3* to give rise to autism.

## Methods

### *C*. *elegans* genetics

*C*. *elegans* strains were cultured and maintained on nematode growth medium (NGM)-agar plates using standard methods at 20°C (Brenner, 1974). The following alleles were used in this study: wild-type N2, *rpm-1(ok364)*, *glo-4(ok362)*, *fsn-1(gk429)*, *unc-2(e55)*, *unc-36(e251)*, *egl-19(n2368)*, *egl-19(n582)*, *egl-19(syb1243)*, *cup-5(ar465)*, *epg-7(tm2508)*, *wdfy-3(ok912)*. Unless otherwise noted, double and triple mutants were constructed following standard procedures, and were confirmed by the associated phenotypes and by PCR/sequence genotyping.

### Transgenic fluorescent markers

The *muIs32* transgene was obtained from the CGC and encodes *Pmec-7*::*gfp + lin-15(+)* [76] and was used to observe the PLM axon. The *jsls973* and *jsls821* transgenes were obtained from Michael Nonet. The *jsls973* transgene encodes *Pmec-7*::*rfp* [45] and was used to observe the PLM axon. The *jsls821* transgene encodes *Pmec-7*::*gfp*::*rab-3* [44] and was used to observe the localization of chemical synapses in the PLM axon. The *yadIs12* transgene was obtained from Dong Yan and encodes *Pmec-4*::*GFP*::*unc-9* [46] and was used to observe electrical synapses in the PLM axon. The *egl-19(syb1243)* mutation was obtained from SunyBiotech. The *cueEx17* and *cueEx18* transgenes were created by injecting *Pmec-7*::*unc-36*::*rfp at 5 ng/ul + Pstr-1*::*gfp at 50 ng/ul*. The *cueEx19 and cueEx20* transgenes were created by injecting *Pmec-7*::*egl-19(gof)* at 5 ng/ul + *Podr-1*::*rfp at 50 ng/ul*. The *cueEx21* transgene was created by injecting *Pmec-7*::*egl-19(gof)* at 25 ng/ul + *Podr-1*::*rfp at 50 ng/ul*.

### Analysis of phenotypes

For analysis of axon termination phenotypes, animals were mounted on a 5% agarose pad and observed with a 40x objective. For PLM axon termination, an axon was scored as defective if it grew anterior to the ALM cell body. PLM neurons were visualized with the *muIs32* transgene which encodes *Pmec-7*::*gfp* and is expressed in all mechanosensory neurons.

For analysis of the PLM chemical synapses, a *Pmec-7*::*gfp*::*rab-3* transgene that expresses the RAB-3 synaptic vesicle marker in the touch receptor neurons was used to visualize synaptic vesicle clusters [44]. The size of each synaptic cluster was measured as previously described [77]. For analysis of PLM electrical synapses, a *Pmec-4*::*gfp*::*unc-9* transgene was used to express the UNC-9 innexin fused to GFP in the touch receptor neurons [46].

For analysis of mechanosensation, we adopted an eyelash touch assay [78]. We assayed gentle touch responses by touching the lateral side of animals with an eyebrow hair. Each animal was subjected to five touches alternating between the anterior and posterior ends and scored by the number of responses elicited. Assays were performed blind to genotype. Three independent samples of 20 animals each were collected by three independent observers and reported as mean percentage scores.

### Ethics statement

Because *C*. *elegans* are invertebrate animals they do not require review by an ethics committee.

## Supporting information

S1 Fig Model for function of VGCCs in relation to the RPM-1 pathway. This hypothetical model could explain the genetic interactions observed between VGCC genes and genes that encode members of the RPM-1 pathway. In this model, VGCCs function with WDFY-3 to negatively regulate an unknown protein (labeled as X). Protein X functions with RPM-1 to promote signaling events downstream of FSN-1 that promote axon termination. Loss of VGCC function causes an increase in protein X function. The additional protein X function works with RPM-1 to promote axon termination mechanism B, thereby compensating for loss of FSN-1 function. Loss of VGCCs does not suppress loss of RPM-1 function because the function of protein X requires RPM-1.
(TIF)

S2 Fig PPM-1 is a protein that functions with RPM-1 to promote signaling events downstream of FSN-1 that promote axon termination. It is possible that PPM-1, or another protein with a similar role, could be protein X (see S1 Fig). Both FSN-1 and PPM-1 promote axon termination by functioning with RPM-1 to negatively regulate the DLK-1 MAP kinase pathway. Thus, it is possible that VGCCs and WDFY-3 could negatively regulate PPM-1, or a protein with a similar role. The extra PPM-1 function could enhance negative regulation of the DLK-1 pathway, thereby compensating for loss of FSN-1 function.
(TIF)

S1 Data Excel file containing the numerical data for Figs 1–5. (XLSX)
