# Supplementary figures and images for: An autism-causing calcium channel variant functions with selective autophagy to alter axon targeting and behavior
Source: PLoS Genet. 2019 Dec 5;15(12):e1008488. doi: 10.1371/journal.pgen.1008488 (PMC6894750; doi:10.1371/journal.pgen.1008488)

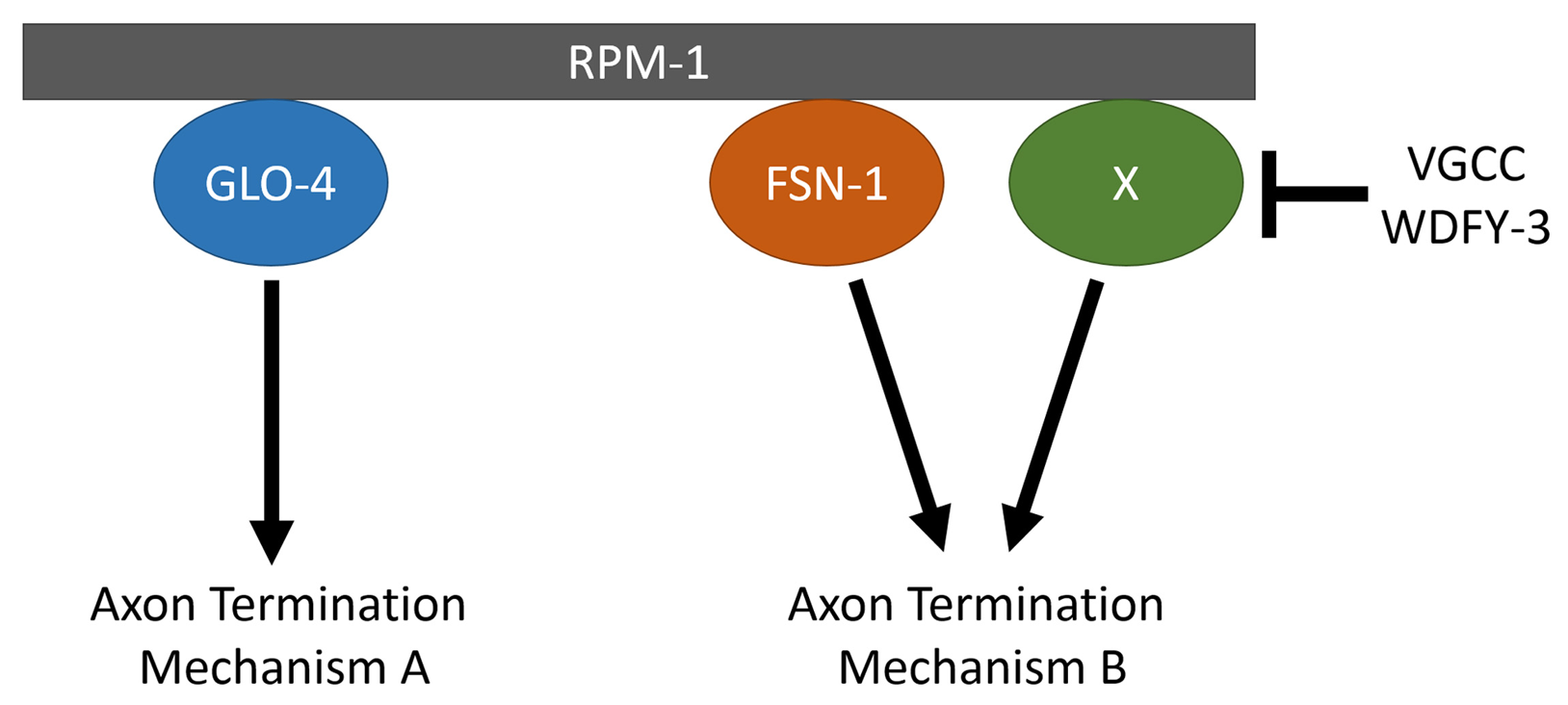

Supplement: S1 Fig — This hypothetical model could explain the genetic interactions observed between VGCC genes and genes that encode members of the RPM-1 pathway. In this model, VGCCs function with WDFY-3 to negatively regulate an unknown protein (labeled as X). Protein X functions with RPM-1 to promote signaling events downstream of FSN-1 that promote axon termination. Loss of VGCC function causes an increase in protein X function. The additional protein X function works with RPM-1 to promote axon termination mechanism B, thereby compensating for loss of FSN-1 function. Loss of VGCCs does not suppress loss of RPM-1 function because the function of protein X requires RPM-1. (TIF) [file pgen.1008488.s001.tif]

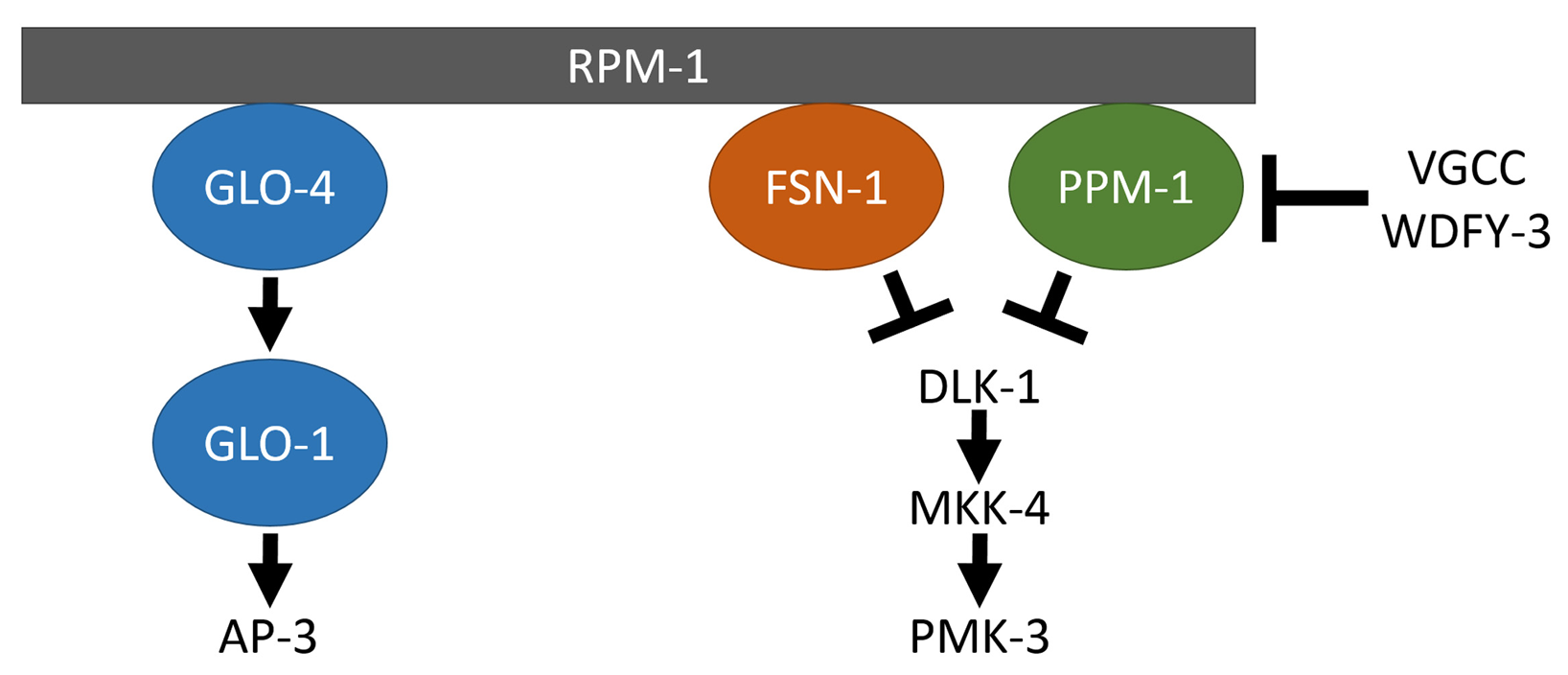

Supplement: S2 Fig — It is possible that PPM-1, or another protein with a similar role, could be protein X (see S1 Fig). Both FSN-1 and PPM-1 promote axon termination by functioning with RPM-1 to negatively regulate the DLK-1 MAP kinase pathway. Thus, it is possible that VGCCs and WDFY-3 could negatively regulate PPM-1, or a protein with a similar role. The extra PPM-1 function could enhance negative regulation of the DLK-1 pathway, thereby compensating for loss of FSN-1 function. (TIF) [file pgen.1008488.s002.tif]
